# An evaluation of the cost-effectiveness of booklet-based self-management of dizziness in primary care, with and without expert telephone support

**DOI:** 10.1186/1472-6815-9-13

**Published:** 2009-12-29

**Authors:** Lucy Yardley, Sarah Kirby, Fiona Barker, Paul Little, James Raftery, Debbie King, Anna Morris, Mark Mullee

**Affiliations:** 1 School of Psychology, University of Southampton, Highfield, Southampton, UK; 2 Department of Audiology, King Edward VII Hospital, Windsor, UK; 3 School of Medicine, University of Southampton, Highfield, Southampton, UK; 4 Hearing and Balance Centre, Institute of Sound and Vibration Research, University of Southampton, Highfield, Southampton, UK

## Abstract

**Background:**

Dizziness is a very common symptom that often leads to reduced quality of life, anxiety and emotional distress, loss of fitness, lack of confidence in balance, unsteadiness and an increased risk of falling. Most dizzy patients are managed in primary care by reassurance and medication to suppress symptoms. Trials have shown that chronic dizziness can be treated effectively in primary care using a self-help booklet to teach patients vestibular rehabilitation exercises that promote neurological adaptation and skill and confidence in balance. However, brief support from a trained nurse was provided in these trials, and this model of managing dizzy patients has not been taken up due to a lack of skills and resources in primary care. The aim of this trial is to evaluate two new alternative models of delivery that may be more feasible and cost-effective.

**Methods/Design:**

In a single blind two-centre pragmatic controlled trial, we will randomise 330 patients from 30 practices to a) self-help booklet with telephone support from a vestibular therapist, b) self-help booklet alone, c) routine medical care. Symptoms, disability, handicap and quality of life will be assessed by validated questionnaires administered by post at baseline, immediately post-treatment (3 months), and at one year follow-up. The study is powered to test our primary hypothesis, that the self-help booklet with telephone support will be more effective than routine care. We will also explore the effectiveness of the booklet without any support, and calculate the costs of treatment in each arm.

**Discussion:**

If our trial indicates that patients can cost-effectively manage their dizziness in primary care, then it can be easily rolled out to relieve the symptoms of the many patients in primary care who currently have chronic, untreated, disabling dizziness. Treatment in primary care may reduce the development of psychological and physical sequelae that cause handicap and require treatment. There is also the potential to reduce the cost to the NHS of treating dizziness by reducing demand for referral to secondary care for specialist assessment and treatment.

**Trial Registration:**

ClinicalTrials.gov trial registration ID number: NCT00732797

## Background

Dizziness has a prevalence of up to 25% in the community [1-4], and 1 in 10 working age adults report some degree of handicap due to dizziness [3]. Dizziness is a more severe problem for older people; more than 1 in 5 people aged over 60 have current dizziness that has led to significant disability, medical consultation or medication use [5]. Dizziness is also associated with falls, fear of falling and loss of independence in older people [6,7].

The most common cause of dizziness in primary care is peripheral vestibular disorder, and serious sinister pathology in patients with no other symptoms is very rare [2,8-12]. Most patients are therefore managed in primary care [1,9,13,14] by reassurance and medication for symptomatic relief [9,11,13,15,16]. However, living with chronic dizziness often entails avoiding all physical activities and situations that might provoke dizziness, leading to clinically significant limitations in physical functioning, reduced quality of life, emotional distress, loss of fitness, unsteadiness and lack of confidence in balance (especially as patients get older), and vulnerability to falling [17-20]. Anxiety about symptoms can contribute to this vicious cycle of chronic dizziness and distress, and a significant minority of patients develop secondary symptoms of panic and agoraphobia [21,22]. Moreover, reviews of the management of dizziness have concluded that no medication has well-established value or is suitable for long-term use, and vestibular rehabilitation (VR) is now recommended as the treatment of choice [11,16,23-27].

VR involves the patient carrying out graded exercises for 10-20 minutes daily for 6 to 12 weeks, consisting of specific eye, head and body movements that stimulate the vestibular system so as to promote neurological adaptation to the altered input from the damaged labyrinth [15,28,29]. Performing these exercises also helps patients to overcome fear and avoidance of activities that provoke dizziness, and regain skill and confidence in balance, reducing the risk of falls [30,31]. Small scale studies have provided some evidence that VR may be an effective treatment for dizziness due to a variety of conditions, including peripheral and central vestibular disorder, benign paroxysmal positional vertigo, whiplash-associated dizziness, acute vertigo, surgery for acoustic neuroma, anxiety, head injury, and dizziness in the elderly [32-40]. A Cochrane review of 21 randomised clinical trials of VR for dizziness due to a range of unilateral peripheral vestibular disorders reported that almost all the trials included in the review demonstrated that VR was a more effective treatment for dizziness than other interventions [25].

Access to VR in the UK usually involves referral to secondary care for a consultation and neuro-otological assessment, often followed by referral to a physiotherapist for VR. Between 9% and 19% of dizzy patients are currently referred to secondary care [1,13]. Two trials, however, have shown that chronic dizziness can also be treated effectively in primary care, using a self-help booklet that instructs patients how to carry out VR with brief support from a trained nurse [41,42]. A non-blind randomized controlled trial provided a preliminary positive evaluation of the efficacy of providing the booklet together with two visits from a trained nurse [41]. The second study was a single blind randomised pragmatic trial of the effectiveness of the booklet, supported by a single session of rehabilitation delivered by nurses working in primary care and two follow-up phone calls [42]. Patients with chronic dizziness from 20 practices were randomised to VR (n = 83) or usual medical care (n = 87). Post-treatment, improvement in the treatment group was significantly greater than in the usual medical care group on all primary outcome measures (including reported symptoms, disability and handicap and objective measurement of postural stability), and was maintained at six months; 67% of treated patients reported clinically significant improvement, compared with 38% controls (relative risk = 1.78, 95% C.I. 1.31 to 2.42). Nearly four times as many treated patients as controls had no provoked symptoms at all. These results are comparable to those achieved in secondary care [25,27].

A further study has investigated whether the booklets are effective if no support is provided [43]. The study was carried out in a sample of people with Ménière's disease, a severe and recurrent form of vestibular disorder for which VR can only provide temporary relief from residual dizziness in between spontaneous attacks of symptoms. Nevertheless, at 6-month follow-up, 45 (37.5%) of the VR group reported improvement compared with 19 (15.8%) controls; the relative probability of improvement compared with controls was 2.37 (95% C.I. 1.48 -3.80). Post-treatment, the rehabilitation group had reduced symptoms, anxiety, handicap, and negative beliefs about dizziness, while the control group showed no improvement. However, reported adherence levels were low and strongly related to outcome. Giving patients the means to manage their symptoms of dizziness in primary care is consistent with the Department of Health initiative to encourage self-care [44] where this can bring benefit to patients and savings for the NHS.

### Rationale and aims

Despite the consensus that VR is the treatment of choice for patients with dizziness of peripheral vestibular origin [23,27], few eligible patients are currently offered VR. Only 2% to 13% of dizzy patients seen by GPs eventually receive VR, of whom around 70% are seen in ENT [1,13]. Yardley and colleagues [42] found that only 3% of participants (5/170) had previously been offered VR, despite a mean duration of dizziness of 8 years, yet 2/3 of participants benefited from it. Although it has been demonstrated that the self-help booklet is effective when combined with support from a practice nurse, this model of managing dizzy patients has not been taken up in primary care due to a lack of skills and resources. The primary aim of the current trial is to determine whether VR can be delivered to dizzy patients in primary care in a feasible, safe and cost-effective way. Two new alternative models of delivery of VR in primary care will be evaluated that may be more feasible and cost-effective.

The first model will examine whether using the self-help booklet with telephone support from a vestibular therapist can be effective. This model of delivery is more feasible than the previous model to roll out across the NHS, as there are already many trained vestibular therapists currently providing therapy in secondary care, whereas very few general practices can provide expert support for VR. Patients may also benefit from having support from an expert vestibular therapist rather than a nurse with limited training. However, remote support from an unknown person might be less effective than close contact with a familiar nurse. The cost of referring patients to secondary care for diagnostic evaluation prior to VR is substantial. The cost varies widely depending on the procedures undertaken, but for patients referred for VR are likely to average around £250 (this figure is based on typical costs of a medical consultation with a senior hospital doctor [45], and the cost of a vestibular assessment and one session of rehabilitation). Expert telephone support for VR can be provided for around £40 an hour (since typical costs for physiotherapists and similar health professionals are no more than £35/hour [45]). Consequently, using our new proposed model of delivery, it should be possible to provide six primary care patients with VR with expert telephone support for less than the cost of referring one patient to secondary care for VR. We predict that by providing treatment and expert support in primary care, the number of patients requiring referral will be reduced, resulting in a lower cost of managing dizziness.

Having previously demonstrated that the self-help booklet alone has a small effect on symptoms and handicap in people with Ménière's disease [43], our second model predicts that it will also be effective in a primary care sample of patients with less severe, recurrent symptoms. In the previous trial, over a third of patients were able to successfully self-treat using the booklet without support. The cost of providing only the self-help VR booklet to patients in primary care is negligible (less than 50 p), and is comparable to the cost of a single prescription of the types of medication commonly used for dizziness symptoms. Since this is a very low cost model of delivery it may well prove cost-effective if a similar proportion of patients benefit from it, and even if only a small proportion of patients can be managed in primary care by the self-help booklet alone, this may still result in substantial savings for the NHS. Because the effect size of using the booklet alone is likely to be small we are unable to power the trial for a definitive test of this hypothesis, but this study will allow us to estimate the likely treatment effect size in a primary care population (which has not yet been established), which would permit a definitive trial in the future.

Provision of the self-help booklet (either with or without telephone support) may therefore represent a cost-effective first stage of patient management, reserving referral to secondary care for patients who require further assessment and therapeutic input.

• Hypothesis 1: Provision of a self-help booklet teaching VR exercises, with up to one hour of telephone support from a vestibular therapist, will be a) more effective than routine care in reducing symptoms (and therefore also disability and handicap) in dizzy patients in primary care, and b) less costly than routine care of dizzy patients.

• Hypothesis 2: Provision of a self-help booklet teaching VR exercises, without any other support, will be a) more effective than routine care in reducing symptoms (and therefore also disability and handicap) in dizzy patients in primary care, and b) less costly than routine care of dizzy patients.

## Methods/Design

### Design overview

**Figure 1:**
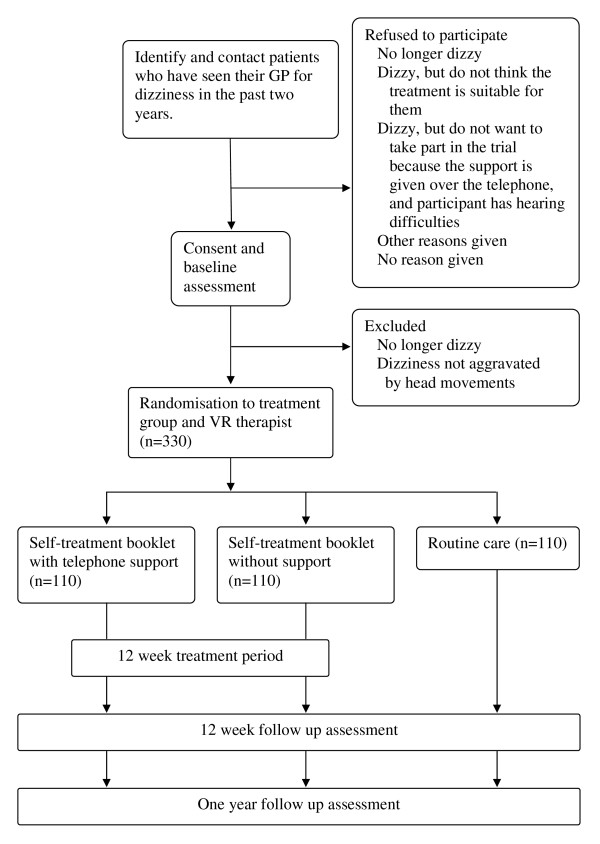
**Flow diagram of the trial design**.

A single blind two-centre pragmatic randomized controlled trial design will be used to compare the effectiveness and cost-effectiveness of a) the self-help booklet with expert telephone support and b) routine care (see Figure 1 for flow diagram of the trial design). We will also evaluate the effectiveness and cost-effectiveness of providing the self-help booklet alone compared with routine care. Participants who receive the self-help booklet (with and without support) will be asked to carry out the exercises in the booklet for up to 12 weeks. We will calculate the total costs of treatment in each arm, including costs of the interventions, primary care consultations and treatment, secondary care consultations, assessment and treatment. Symptoms, disability and handicap will be assessed by validated questionnaires at baseline, 12 weeks after allocation to treatment group, and at one year follow-up. We will compare the change in symptoms and quality of life and the total costs of treatment in each group of patients. This design was chosen so it can be compared with a previous successful trial [42], and if found to be effective, these models of delivery can be easily rolled out through the NHS across the region and the UK.

### Setting

The study will be carried out in 30-50 general practices around two centres (the department of Audiology at King Edward VII Hospital in Berkshire and the University of Southampton), to sample differing existing models of delivery of routine primary and secondary care. These general practices will be sampled to include urban, suburban and rural practices with varying levels of social deprivation. Telephone support will be provided by vestibular therapists based at the two centres, and data will be collected centrally at the University of Southampton.

### Sample size/power calculation

Assuming that we will achieve a treatment effect at least as great as that in our last primary care trial (d = .41) [42], we will need 95 patients per group to test a two-tailed hypothesis with 80% power and 5% significance level. Drop-out at six months in our previous primary care trial was 8.8% (and only 4.7% in our previous self-treatment trial), but since we have a longer follow-up we will allow for 15 patients to drop out of each condition (14.25%), giving a sample size of 110 in each condition (a total of 330).

This sample size should be attainable in practice, as the two previous primary care trials using similar recruitment methods and recruitment time scales (15 months) were able to attain comparable sample sizes. Yardley and colleagues [41] identified 327 eligible patients from 10 general practices of varying size, from which 115 were recruited into the trial. Our last primary care trial [42] also followed the same time scale and recruitment method, contacted approximately 680 participants at 20 general practices and recruited 170 participants. Based on these numbers we would expect to contact between 981 and 1700 patients from 30-50 general practices to recruit approximately 255 - 575 participants into the trial.

### Participant recruitment and selection

Potential participants will be identified by practice staff (with the help of a research associate who will hold honorary contracts with the PCTs) searching the computerised database of the practice. The computerised database search will follow inclusion and exclusion screening criteria that proved successful in previous primary care studies of dizziness to safely and effectively identify patients likely to benefit from the self-help exercises [41,42]. We have support for patient recruitment from the Primary Care Research Network South East, South West and Western branches, and will therefore receive service support costs for general practices taking part. Patients can also be directly identified by their general practitioner, and posters will be displayed in practices to inform patients that they can ask to participate.

Eligible patients will be sent a letter and invitation pack (including the information sheet and consent form) from their GP inviting them to take part. Patients who do not wish to take part in the trial but are happy to provide further information are invited to complete a trial refusal slip indicating whether they are a) no longer dizzy, b) dizzy but feel that the treatment would not be suitable for them, c) dizzy but do not want to take part in the trial because the support is given over the telephone, and the patient has hearing difficulties, or d) do not want to take part in the trial for some other specified reason. Patients who have consented to take part in the trial will receive a copy of their signed consent form (also signed by the research associate). The research associate will also keep a copy for the trial file, and then send the original to the patient's GP practice for inclusion with the patient's medical notes. After baseline questionnaire assessment, patients will be excluded prior to randomization if they report that they are no longer dizzy, or their dizziness is not aggravated by performing head movements (in which case their dizziness is not typical of vestibular imbalance and is unlikely to respond to the self-help exercises). All respondents will receive a group allocation letter, explaining which treatment group they have been assigned to, or explaining that they have not met the inclusion criteria.

#### Inclusion criteria

a) Patients must be 18 or over and registered at a participating general practice.

b) Patients must have seen their GP for dizziness some time in the past two years (Participating practice computerised databases will be searched using search terms such as: vertigo; dizziness; Meniere's disease; balance problems; vestibular; prochlorperazine; cinnarizine; betahistine; diuretics - terms used will vary according to how patient data is recorded by the practice).

c) Patients must return the completed and signed consent form

d) Patients must indicate in the baseline questionnaire assessment that they still experience dizziness and that quick head movements make them dizzy.

#### Exclusion criteria

a) Non-labyrinthine cause of dizziness identified by GP (in which case patients will not benefit from the vestibular rehabilitation exercises)

b) Medical contraindications for making normal head movements (e.g. severe cervical disorder), in which case patients will be unable to carry out the exercises.

c) Serious co-morbidity (e.g. progressive central disorder, terminal illness, and moderate to severe dementia or mental illness).

d) Moved away from practice

e) Recently deceased

f) Non-English speakers and people unable to read and write English.

### Randomization to treatment groups and blinding

Eligible patients who have signed a consent form to take part will be randomised by an administrative assistant at the University of Southampton with no prior contact with the patient. The assistant will use a remote randomisation service, such as the Trans European Network for Clinical Trials Services (TENALEA), to generate a schedule for assigning participants on the basis of block randomization within practice (block size 9) after stratification for severity of symptoms (score below 12 or 12 and above) on the main outcome measure at baseline. To ensure that centre effects are not confounded with therapist effects, patients will be randomised to the three treatment groups and to the three therapists. The research associate collecting the data will remain blind to patient allocation until after the 12 week follow up. The research associate will then become unblinded to the name and group allocation of a small subsample of participants who are taking part in interviews of their experiences of using the self-treatment booklets and telephone-based therapist support. The research associate will not become unblinded to these participant's trial number, which is the only identifier by which outcome data are identified. The statistician analysing the data will remain blind to patient allocation throughout the trial. Blinding of patients is not possible due to the nature of the therapy.

### Trial treatment groups

Participants will be randomly assigned to receive a) the self-help booklet with telephone support (1 × 30 minutes followed by 2 × 15 minutes), b) the self-help booklet without support, or c) routine care. Participants who receive the self-help booklet will be asked to carry out the exercises in the booklet for 10 minutes, twice a day, every day for up to 12 weeks. Participants in all three treatment arms will be advised in the information sheet and group allocation letter that they remain free to seek any other consultations or treatment they feel they need while participating in the trial. Participants will therefore receive, with their group allocation letter, a participant diary to help them to keep track of the consultations and health care they receive for dizziness related symptoms over the forthcoming year.

#### a) Self help booklet with telephone support

The 110 participants randomised to receive the self-help booklet with telephone support will receive a self-help booklet and an invitation (by telephone) from the administrative assistant to arrange a convenient time for a 30 minute telephone call from one of two qualified vestibular therapists with experience of treating dizziness (FB and AM), or a trainee vestibular therapist. The therapist will talk participants through the booklet, answer questions and concerns and advise participants how to use the booklet for their particular problems and needs. This session will be followed up by two 15 minute phone calls one week and three weeks after starting self-help, to ask about how treatment has been progressing and to advise on how to overcome any problems, and how to make the exercises more challenging as their symptoms improve. Telephone support will be standardised across the three therapists using the half-day training workshop and session checklist employed in our previous trial [42]. Combined with remote telephone delivery, standardisation should ensure that therapist effects on outcome are minimal.

The self-help booklet explains in lay terms the causes of symptoms and why VR should help to resolve them. Details are given of daily balance training exercises (that involve gently increasing the speed of making normal head movements) to carry out in the home and how to tailor these to the particular symptoms experienced by the individual patient. Participants are expected to carry out the exercises for 12 weeks (by which time most recovery that is likely to be achieved will have occurred), or until they are asymptomatic (if this occurs in less than 12 weeks). Advice is also given on resuming activities in daily life that may have been avoided because of dizziness. To ensure positive but realistic beliefs, a question-and-answer format is used to provide evidence of treatment relevance and efficacy and to address common concerns. To promote confidence and adherence, the booklet helps patients to make a specific graded goal plan and written commitment and to adapt the intervention to their symptoms, capabilities, and lifestyle. The booklet also provides advice on how to monitor treatment (using a standard self test and chart to select appropriate exercises each week), and highlights symptoms that indicate the patient should cease exercising and seek medical advice.

#### b) Self help booklet without support

The self-help booklet has been designed and evaluated so that it can be used with or without support. Therefore, participants randomized to receive the booklet with no support will simply be sent the booklet to read and follow without advice (but will be informed that if they have concerns about the safety of exercising or experience adverse symptoms from exercising they should contact the research team for expert advice).

#### c) Routine care

Participants in the routine care arm will be managed as normal within that practice. After the one year follow up, participants allocated to the routine care condition will be sent the self-help booklet on request.

### Post-trial arrangements

After the trial has ended, those in the self-help booklet groups can keep the self-help booklet they have been given. A self-help booklet will be sent to those in the routine care group if they wish to receive one. As this study is a trial, the telephone advice and support from the vestibular therapists will not be available beyond the 12 week treatment time outlined above for those in the self-help booklet with telephone support group, and will not be available for those in the other groups (this is stated in the Information Sheet). All groups will continue to receive normal medical care for the treatment of their dizziness. At the end of the project, a lay summary of the results of the trial will be sent to all the participating practices, and to patients on request.

### Management of trial fidelity

The design of the trial is intended to minimise centre, therapist and practice effects on outcome. Randomisation is at the level of the individual participant rather than practice, since contamination is improbable (in theory, participants receiving the booklet could show it to those who did not receive it, but it is highly unlikely that they would come into contact and discuss the trial and treatment). Practice effects are further minimised by detailed guidance from the research team regarding inclusion and exclusion criteria for patients to be invited to participate. Since there will be numerous referring practices from each centre, centre effects on clinical outcome should not be significant.

Although the trial intervention is within the routine professional competence of the three VR therapists, therapist effects are minimised by strict manualisation of treatment. The VR booklet forms the basis of treatment, and the therapist's role is simply to ensure that the patient understands and follows the booklet correctly, and to answer questions and respond to concerns. Telephone support will be standardised using a written workshop format with example patient case scenarios published with the paper reporting the previous trial [42]. The content of each therapist session is specified by a checklist. All telephone support sessions will be tape-recorded, and the research associate will examine treatment fidelity by comparing therapist checklists with a sample of taped sessions to determine the accuracy of reporting completion of the treatment protocol. Therapist effects are also likely to be minimised by the absence of personal contact or an ongoing professional relationship between the therapist and patient.

### Potential benefits and adverse effects

The trial has several anticipated benefits as well as predictable risks and inconveniences for patients taking part. Previous research has shown that chronic dizziness can be treated effectively using the self-help booklet, although we cannot guarantee that the self-help will be effective for all patients. Benefits may also include feeling that they have helped a medical research project. We hope the results will be used to review current primary care services for people with dizziness and this may result in improved services though there is no guarantee of this. Risks and inconveniences include the time and effort required to carry out the self-help exercises and complete the questionnaires (approximately 20-25 minutes for each stage of the trial). In addition to this, the self-help exercises require patients to deliberately make themselves dizzy in a controlled manner. Although some people may find this unpleasant, the exercises cannot cause any damage to their balance system.

#### Stopping rules

Since the exercises do not require any unusual or strenuous movements, the risks of carrying them out are very slight. Nevertheless, there is a remote possibility that the exercises could bring on symptoms that could signal the potential for serious medical consequences, specifically cervical damage, damage to the inner ear (perilymph fistula), or vertebrobasilar ischaemia. These symptoms, which are detailed in the information sheet and in the self-help booklet, are:

• A sharp, severe or prolonged pain in the neck, head or ear

• A feeling of fullness in the ear

• Deafness or noises in the ear

• Fainting with loss of consciousness or blacking out

• Double vision

• Numbness, weakness or tingling in the arms or legs

In the very unlikely event that a participant experiences these symptoms, they are advised (in the information sheet, group allocation letter and self-help booklet) to stop the exercises immediately, and consult their vestibular therapist if in the telephone support group, or the research associate if in the booklet without support group. Problems are usually managed by advising participants to do the exercises more slowly and gently (i.e. usually the problems are just some non-serious aggravated neck pain or dizziness). If thought to be necessary, participants will be referred to their GP; however, this has not been necessary in our previous clinical trials, and other clinical trials of VR have also reported no adverse effects as a result of undertaking VR [25].

### Data collection and follow up

Participants in all three treatment arms of the trial will be sent, by post, questionnaire packs (and a freepost return envelope addressed to the research associate) to complete at baseline, 12 weeks after being allocated to a treatment group, and at one year follow up. The baseline and one year follow up questionnaires should take approximately up to 20 minutes to complete, and the 12 week follow up questionnaire should take approximately up to 25 minutes to complete.

Self-report of service use (GP visits, medication, referral etc.) will be checked by the research associate for accuracy against the medical records in a random sub-sample of at least 20 percent of patients. If significant discrepancies between records and patient reports of service use are found then a larger proportion will be checked if time permits.

To incorporate representation of patients' views of the trial into our evaluation, telephone-based interviews will be conducted with 24 - 30 participants; 12 - 15 participants from the booklet only condition and 12 - 15 from the booklet with therapist support condition. These will take place after completion of the 12 week questionnaire, and will last approximately 30 minutes. The interviews will explore participants' experiences of using the self-treatment booklets and telephone-based therapist support.

#### Non-responders and drop out from the trial

Non-responders to the baseline, 12 week and one year follow up questionnaires will receive one postal reminder after 3 weeks, and a follow-up telephone call (with collection of data by phone interview if preferred) for those who still do not respond after a further 3 weeks. Dizzy patients who volunteer to participate are typically well-motivated to adhere to trial procedures; in our last trial we used postal measurement of outcomes, and only 17 patients out of 360 failed to complete the final follow-up [43]. Participants who have chosen to drop out of the trial are asked in the reminder letter to return the questionnaire unanswered, so that we know that they do not want receive the telephone reminder or further information about the trial. Participants can either withdraw from the trial entirely, or may choose to withdraw from carrying out the exercises in the self-treatment booklet but still continue to complete the follow up questionnaires. Participants remain free to drop out of the trial at any stage, and will not be replaced if drop out occurs after randomisation. Follow-up by telephone of non-respondents will be carried out by the blinded research associate; patients will be advised not to reveal their trial status, and any failure of blinding will be recorded and potential effects on trial outcomes analysed.

### Outcome measures

The measures used at each data collection point are summarised in Table 1. For comparability with the previous trials of the self-help booklet, the primary outcome measure will be self-reported symptoms of dizziness, assessed by the Vertigo Symptom Scale - Short Form [46], which assesses frequency of fifteen dizziness-related symptoms during the past month on a five point scale. A recent study has suggested that the Vertigo Symptom Scale - Short Form has a stable and reliable two factor structure, relating to vertigo and autonomic anxiety [47]. As it is of clinical interest to be able to discriminate between symptoms resulting from vertigo and secondary symptoms due to autonomic anxiety, these subscales will therefore be explored as secondary outcomes.

**Table 1:** Measures used in baseline, 12 week and one year follow-up questionnaires.

Measure	Baseline	12 week	One year
Demographic characteristics	X		
Duration of current symptoms	X		
Diagnosis received	X		
Dizziness due to head movements	X		
Vertigo Symptom Scale - Short Form (VSS-SF)	X	X	X
Dizziness Handicap Inventory (DHI)	X	X	X
Hospital Anxiety and Depression Scale (HADS)	X	X	X
Management of dizziness (consultations in primary and secondary care and other sources; hospital stays; medication/treatment; days off work)	X		X
Quality of Life (EuroQoL - EQ-5D)	X	X	X
Your dizziness or unsteadiness now		X	X
Problematic Experiences of Therapy Scale (PETS; booklet arms only)		X	
Adherence to therapy (booklet arms only)		X	
Total time spent on each exercise (booklet arms only)		X	
Continuation of therapy (booklet arms only)			X

Secondary analyses will also include two less precise and sensitive but clinically meaningful measures of symptom improvement. Clinically significant change on the primary outcome measure questionnaire will be examined, as in the last study, by identifying the proportion of patients with a symptom score reduction of at least three points. Subjective improvement in health will also be assessed by a single item ('your dizziness or unsteadiness now') used in the previous trials, asking whether, during the past week, the participant felt better, much the same, or worse than when completing the baseline assessment. This item provides a simple patient-generated estimate of effectiveness, corresponds well to other outcome measures, and ensures some follow-up data is obtained for almost all participants, since patients will usually respond to this item on the telephone even if they do not wish to fill in a questionnaire.

Additional secondary outcomes that we will assess will be quality of life effects of dizziness, and anxiety and depression. As in our previous studies, these will be assessed by the Dizziness Handicap Inventory [48] and the Hospital Anxiety and Depression Scale [49]. We will also assess quality of life using the EuroQoL - EQ-5D [50], as this provides data that can be compared with other conditions and treatments. For the cost-effectiveness analyses, at one year follow-up we will gather details from the medical records and ask patients for retrospective reports for the past year of dizziness-related: number of GP visits; medication taken; days off work; secondary referrals. The cost and outcome data will be combined to estimate the incremental cost per QALY of the two interventions from an NHS perspective. Post-treatment we will assess adherence to therapy in conjunction with the previously validated Problematic Experiences of Therapy Scale (PETS) [43], and at one year follow up we will also ask participants whether they have continued to use the VR exercises. At baseline we will also record demographic characteristics (age, sex, years of education), duration of dizziness, diagnosis, and previous management of dizziness (e.g. medication, referrals).

### Statistical analysis

Data entry will be carried out by the administrative assistant and double checked by the research associate. The trial will be analyzed on an intention to treat and per protocol basis, using SPSS for Windows (SPSS Inc., Chicago) and Stata (StataCorp). Levels of missing data will be investigated, but are expected to be very low for the primary outcome (see above). Missing data on individual items on scales used to measure secondary outcomes will be replaced by the blinded research associate by the mean for that individual on that scale, provided that the majority of items have been completed. Where relevant, outcome variables will be checked for the assumption of normality. If the assumption is not met, data transformations or equivalent non-parametric tests will be conducted. Effect sizes and confidence intervals will be reported for all tests.

Qualitative methods will be used by the research associate to examine treatment fidelity by comparing therapist checklists with a sample of taped sessions to determine the accuracy of reporting completion of the treatment protocol. The research associate will also use qualitative methods to analyse the interviews of participants' experiences of using the self-treatment booklets and telephone-based therapist support.

#### Primary and secondary analysis

Statistical analysis will be carried out by LY and MM at the end of the trial who will both be blind to participants' group allocation. The main analysis will compare patients randomised to the self-help booklet with telephone support versus patients randomised to routine care. The analysis will be performed on the primary outcome symptom score (the Vertigo Symptom Scale - Short Form) at 3 months after randomisation, using ANCOVA (analysis of covariance) adjusted for symptom score at baseline. Continuous outcomes on the secondary outcome measures will also be evaluated using ANCOVA. All continuous outcome variables will be checked for the assumption of normality. If the assumption is not met, bias corrected and accelerated bootstrap methods will be used to construct confidence intervals. Binary outcomes will be compared between the two groups using logistic regression. All analyses will be adjusted for the level of the relevant outcome variable at baseline. All analyses will also be adjusted for any confounding variables that may differ by chance between the two groups at baseline. These analyses will be repeated for outcome at 12 months. Further secondary outcome analyses will repeat the above, comparing outcomes between patients randomised to the self-help booklet without any other support versus patients randomised to routine care.

To check our assumption that centre, therapist and practice effects do not significantly affect our primary outcome, we will use summary measures, forest plots and tests of interaction and heterogeneity. If there is evidence of such effects, we will perform sensitivity analyses by adding random effects terms to the model comparing the two groups. The study is not, however, powered for such tests.

#### Cost -effectiveness analyses

For the cost-effectiveness analyses, the EuroQol - EQ-5D data will be converted to utility values using the published national tariff. Incremental cost effectiveness ratio (ICER) data will be estimated. Cost effectiveness acceptability analysis will quantify the level of uncertainty in stochastic data. Sensitivity analysis will test the structural uncertainty in a simple model. To determine the probability that one intervention is cost effective relative to the other, we will examine bootstrapped estimates of imputed mean costs, the cost effectiveness plane of the incremental cost effectiveness ratio, net benefit statistics and cost acceptability curves. As in the clinical outcome analyses, these analyses will be adjusted to control for the effect of any clinically significant difference between the groups on baseline variables.

### Public involvement in the trial

The principal organization in the UK that represents the interests of people with vertigo and dizziness due to vestibular disorder is the Ménière's Society, a self-help group with charitable status and over 5,500 members. The aims of the Society include providing information and support to members and their families, raising awareness among health professionals and the general public, and encouraging research into the causes and treatment of Ménière's disease. The most distressing symptom for most people with Ménière's disease is vertigo and dizziness. The Research Trustee of the Ménière's Society contributed to the development of this proposal, and representatives of the Ménière's Society will sit on the steering group for the project. The Ménière's Society currently produces and distributes the booklets that will be used in the trial, and will work to disseminate the trial findings and the booklets to people with vertigo and health professionals if the intervention is confirmed as effective.

### Trial management

Each centre team will meet monthly, and the whole team will meet 4 times a year. Because of the geographical dispersion of the team, for rapid communication between meetings email will be most efficient. The research associate will circulate a monthly update to review progress relative to the project plan, highlighting any issues that need to be addressed. Each team member will consult the other team members immediately by email and/or phone on any issues that arise.

### Trial steering committee

Management of the project will be overseen by a steering group including members who are independent of the research team (including the Director and Research Trustee of the Ménière's Society, and a neuro-otological expert), who will scrutinise the progress and conduct of the research annually.

### Ethical approval and data protection

The trial will be conducted, analysed and reported in accordance with the ethical principles described in the Declaration of Helsinki, ICH Guidelines for Good Clinical Practice, and the CONSORT Statements. Ethical approval for the trial has been given by the Southampton and South West Hampshire (B) REC, reference number 08/H0504/31. R&D and data protection approval has also been obtained from the relevant geographical areas. Indemnity cover is provided by the University of Southampton. All patients will receive an information sheet about the trial, and written consent will be obtained from all patients before entering into the trial. Consent will include permission to access relevant sections of participants' medical records, to audio-tape the telephone support sessions, and to inform participants' GPs of their participation in the trial. Participants are informed that they remain free to withdraw from the trial at any time.

All information collected during the course of the research will be kept strictly confidential, although source data will be subject to monitoring, audits and REC reviews. Therefore any information taken from medical records will have the name and address removed when it leaves the general practice, and participants will not be identifiable in any report or publication. Data will be handled, processed, stored and destroyed following procedures in keeping with the Data Protection Act 1998. Data from this study will be kept for 10 years either at the University of Southampton or a secure offsite location, and will then be disposed of securely.

### Reporting and dissemination

We will disseminate the findings of the research to three key populations: lay people with dizziness (who need to be informed about the availability and effectiveness of treatment); primary care professionals (particularly GPs), who could implement provision of VR in primary care; and clinicians currently treating dizziness in secondary care, who could provide expert support for treatment in primary care. We will disseminate our findings to lay people with vertigo and dizziness nationally and internationally through the newsletter and web pages of the Ménière's Society, and through press releases and media interviews. We will disseminate our findings to primary care professionals nationally and internationally by submitting academic papers to journals such as the British Medical Journal and articles to magazines such as Pulse and Doctor. We will disseminate our findings within the region through local postgraduate meetings and ENT seminars for GPs, and by giving reports of our findings to primary care trust managers and practice-based commissioning groups as appropriate. We will also seek to present our findings at regional and national meetings of the Society for Academic Primary Care, and at the North American Primary Care Research Group Conference. We will disseminate our findings to those currently involved in the management of vertigo and dizziness (e.g. clinical scientists in audiology, hearing therapists, physiotherapists, ENT consultants, neurologists, and audiological physicians) by submitting academic papers to journals such as the International Journal of Audiology and Clinical Otolaryngology, and magazines such as ENT News. We will also present talks at relevant regional and national meetings and conferences (e.g. national Balance Interest Group meetings of physiotherapists and clinical scientists in audiology, conferences of the international Barany Society and British Academy of Audiology).

## Discussion

The interventions we propose to evaluate in this trial are both based on a self-help booklet that patients with dizziness can use to carry out rehabilitation. If our trial shows that patients can cost-effectively manage their dizziness in primary care, using a booklet with or without expert telephone support, then these models of delivery can be easily rolled out through the NHS across the region and the UK. The primary outcome would be rapid relief of the symptoms of the large number of patients in primary care who currently have chronic, untreated, disabling dizziness.

The cost of producing the self-help booklet is less than 50 p, so booklets could be very easily provided to any practices that wished to use them. To maximize efficient and safe use of these booklets, patients could be screened by their GP to exclude sinister pathology and contraindications to exercise, provided with the booklet (with or without expert support), and then referred on to secondary care for specialist assessment and treatment only if rehabilitation fails to relieve their symptoms. If the most cost-effective model is to also provide expert telephone support, this can be easily provided by physiotherapists, hearing therapists and clinical scientists in audiology who have the relevant training and experience; there are already large numbers of suitably qualified people, currently working mainly in secondary care settings.

The proposed models of care would further the aims of the NHS Improvement Plan by providing a service based on supported self-management. Demonstration of a cost-effective model for managing dizziness in primary care also has the potential to reduce the cost to the NHS of treating dizziness by reducing demand for referral to secondary care for specialist assessment and treatment. If patients can be treated promptly in primary care they may be less likely to develop psychological and physical sequelae that cause handicap and require treatment.

## Competing interests

The authors declare that they have no competing interests.

## Authors' contributions

All authors participated in the conception and design of the trial. LY, JR, and MM participated in plans for the analysis. LY and SK were involved in drafting the manuscript. All authors read and approved the final manuscript.

## Pre-publication history

The pre-publication history for this paper can be accessed here:

http://www.biomedcentral.com/1472-6815/9/13/prepub
